# Concepts and Applications of Information Theory to Immuno-Oncology

**DOI:** 10.1016/j.trecan.2020.12.013

**Published:** 2021-02-20

**Authors:** Aleksandra Karolak, Sergio Branciamore, Jeannine S. McCune, Peter P. Lee, Andrei S. Rodin, Russell C. Rockne

**Affiliations:** 1 Department of Hematologic Malignancies Translational Science, Beckman Research Institute of City of Hope, Duarte, CA, USA; 2 Division of Mathematical Oncology, Department of Computational and Quantitative Medicine, Beckman Research Institute of City of Hope, Duarte, CA, USA; 3 Department of Computational and Quantitative Medicine, Beckman Research Institute of City of Hope, Duarte, CA, USA; 4 Department of Immuno-Oncology, Beckman Research Institute of City of Hope, CA, USA

## Abstract

Recent successes of immune-modulating therapies for cancer have stimulated research on information flow within the immune system and, in turn, clinical applications of concepts from information theory. Through information theory, one can describe and formalize, in a mathematically rigorous fashion, the function of interconnected components of the immune system in health and disease. Specifically, using concepts including entropy, mutual information, and channel capacity, one can quantify the storage, transmission, encoding, and flow of information within and between cellular components of the immune system on multiple temporal and spatial scales. To understand, at the quantitative level, immune signaling function and dysfunction in cancer, we present a methodology-oriented review of information-theoretic treatment of biochemical signal transduction and transmission coupled with mathematical modeling.

## Introduction

The immune system relies on efficient and accurate transmission of information, from recognition of pathogens/cancer cells to precise orchestration of an immune response, through sequenced activation and suppression mechanisms. At a conceptual level, ‘information’ is defined as the resolution of uncertainty. In the context of immuno-oncology, one would like to trace information as a signal propagates within the immune system – for example, within immune signaling networks and pathways. In order to do that, one needs to start with the quantification of information, by introducing measures and metrics that formalize the notion of resolution of uncertainty. A basic, probabilistic measure is Shannon information [1,2]. For a discrete event *x* with probability *P*, information can be defined as follows:

[1] Information(x)=− log P(x).

Over the years, many characteristics of the immune system have proved amenable to representation and modeling with concepts from information theory (IT) (Box 1) [3–8]. The storage, transmission, encoding, and flow of information between cell populations in the immune system are facilitated by a large repertoire of molecules. As the resolution of biology continues to increase through technological advancements, including single-cell sequencing, high-dimensional flow cytometry, and mass spectrometry, so, too, does our ability to characterize and quantify information flow in the immune system [7–10]. This methodology-oriented literature review of applications of IT to the immune system includes a brief review of fundamental concepts and quantities of IT, applications of IT to the immune system in health and cancer, information flow and signal processing as they relate to biochemical signaling and communication channels within and between immune cells, and a forward-looking perspective on IT applications and new concepts of immune system signaling. This review is aimed at immuno-oncologists interested in IT and computational researchers interested in applications of IT to immuno-oncology.

## Concepts in Information Theory

The concept of information as a crucial component of communication theory was established by Shannon in 1948 and subsequently popularized by Shannon and Weaver (Box 1) [1,2]. It centered on the notion of entropy. Shannon entropy of a discrete random variable *X* with possible outcomes (events) *x* is the average level of information (Equation 1) over all possible outcomes:

[2] $$H\left(X\right)=-\sum_{x\in X}P\left(x\right)\text{ log}\;P\left(x\right).$$

While having its origins in Boltzmann’s statistical mechanics and thermodynamic entropy, Shannon entropy, probabilistic in nature, is not intrinsically bound to a particular physical, chemical, or biochemical phenomenon; consequently, entropy-based methods have been used to quantify disorganization or randomness in many components of biological systems [11–15]. Some examples of quantification of uncertainty associated with the outcomes of *x* can be found in [16], including the ‘classic’ one-bit quantification (using base 2 logarithm) of entropy as the uncertainty associated with binary outcomes, such as the flip of a coin. Differential (or continuous) entropy is an extension of Shannon entropy to the random variables with continuous probability distributions. ‘Mixing and matching’ discrete and continuous variables in the same IT analytical framework is a nontrivial undertaking; we discuss one practical approach (mixed mutual information) later.

Recently, biomedical studies within and beyond the quantitative immunology domain have increasingly dealt with interdependencies; a prominent example is dissecting multiple biomarker dependencies associated with various cancer types, subtypes, and stages. In such cases, another IT-derived concept, mutual information, has proved especially appropriate [17–19]. With mutual information, the amount of information gained by observation of one variable through another variable reflects their mutual dependence, defined as follows (for discrete random variables):

[3] I(X;Y)=H(X)−H(X|Y),

where *X* and *Y* are independent random variables if and only if mutual information *I*(*X*;*Y*) = 0, *H*(*X*) is the entropy of *X*, and *H*(*X*|*Y*) is the conditional entropy (for discrete random variables):

[4] $$H\left(X|Y\right)=-\sum_{x\in X,y\in Y}P\left(x,y\right)\text{ log}\frac{P\left(x,y\right)}{P\left(y\right)}.$$

The concept of mutual information can be extended to a mix of continuous and discrete variables (mixed mutual information) [16,17]. Although mutual information is directly linked to the amount of information (entropy) of each individual variable, mutual information specifically aims at quantification of the information communicated, or shared, between variables [19–21]. As such, applications of mutual information allow quantification of information ‘dialogues’ in systems and networks [22–25].

In order to quantify information across two probability distributions (e.g., cases versus controls, disease versus health, or therapy responders versus nonresponders), a concept of cross-entropy of *P*(*x*) distribution relative to *Q*(*x*) has been proposed [26]. For discrete probability distributions, cross-entropy is defined as:

[5] $$CE\left(P\|Q\right)=\sum_{x\in X}P\left(x\right)\text{ log}\frac{P\left(x\right)}{Q\left(x\right)}-\sum_{x\in X}P\left(x\right)\text{ log}\;P\left(x\right).$$

The first term in Equation 5 corresponds to relative entropy, also known as Kullback–Leibler (KL) divergence, which can be used to estimate the expectation of information gain. The KL divergence measure, and the Jensen–Shannon distance (JSD; symmetrized KL), are commonly used to quantify the distance, or dissimilarity, between two distributions. Similar to other IT measures, cross entropy and distribution dissimilarity measures can be extended to the continuous random variables. In noisy signaling systems, however, it is an unavoidable loss, not gain, of information flowing through the communication channel, an IT-derived model of information flow over time between variables in a dynamic system, that can be further quantified as information or channel capacity (Boxes 1 and 2).

The above notions, measures, and metrics are useful for quantifying information as applied to discrete and continuous random variables and their distributions. Depending on the specific questions and the exact nature of the observed data, IT approaches can be applied to any inverse problem in modern biomedical research (e.g., oncology, genomics, neuroscience), often overlapping with more ‘traditional’ statistical techniques, Bayesian statistics, statistical learning, and machine learning. Selection of the appropriate IT concepts for a given analysis depends not only on the questions asked but also on the fine structure of the biological data (Table 1). Ideally, a variety of methods should be applied to any new biological system/domain/dataset of interest – investigators should ‘mix and match’ available IT, statistical, and machine learning methodology when facing a new domain.

## Applications of Information Theory to Quantify Information in the Immune System

### Entropy as a Measure of System Disorganization

**Figure 1.**
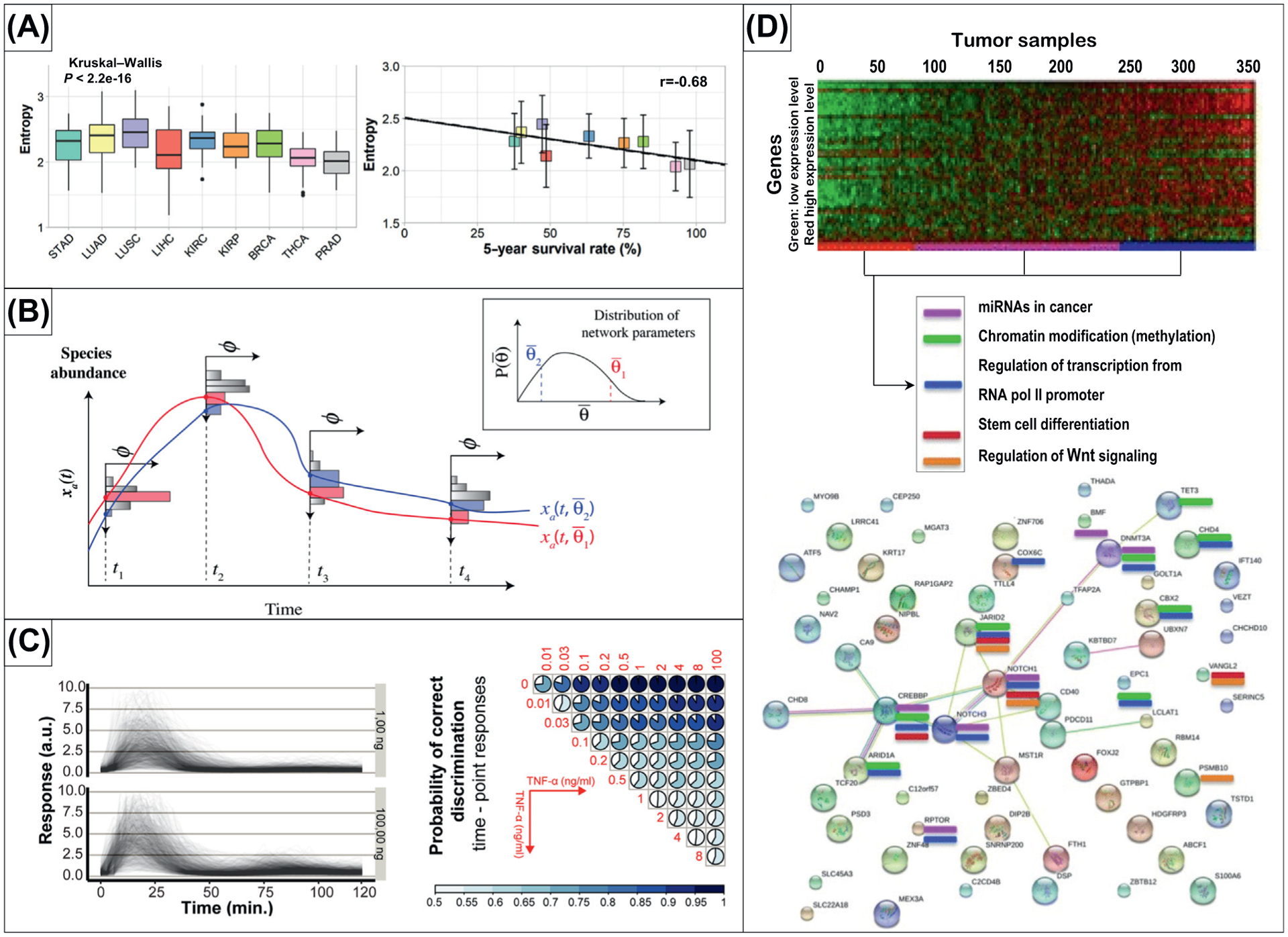
Information-Theoretic Approaches to the Quantification of Information in Immune Responses and in Immuno-Oncology. (A) Left: Box plot of entropy of upregulated gene subnetworks across cancer types.Right: Correlations between entropy and 5-year survival rates from [12]. (B) Illustration of the MERIDIAN inference approach and application of maximum entropy from [29]. (C) Top: Analysis of nuclear factor-κB (NF-κB) responses to tumor necrosis factor alpha (TNF-α) stimulation. Bottom: Probabilities of the correct pairwise discrimination, both from [33]. (D) Applications of CorEx algorithm to discover the associations between genes related to miRNA, chromatin modifications, epithelial-to-mesenchymal transition, increased aggressiveness, and metastasis in breast tumors from gene expression profiles [37]. Reprinted with permission. Abbreviations: a.u., arbitrary units; BRCA, breast invasive carcinoma; LUAD, lung adenocarcinoma; LUSC, lung squamous cell carcinoma; LIHC, liver hepatocellular carcinoma; KIRC, kidney renal clear cell carcinoma; KIRP, kidney renal papillary cell carcinoma; PRAD, prostate adenocarcinoma; STAD, stomach adenocarcinoma; THCA, thyroid carcinoma.

Perhaps the most commonly appreciated concept from IT is that of entropy, often used to quantify randomness or disorganization. Within the immune system, the notion of ‘organization’ has various implications. On the one hand, a functional immune system that is well organized, with the tightly regulated and coordinated interactions between cells and molecules required to mount an immune response, is advantageous. On the other hand, diversity and some degree of randomness may also be advantageous in recognizing a variety of pathogens. Indeed, entropy has been used to quantify both of these aspects of the immune system in thought-provoking and novel ways (e.g., as a foundational rationale for the emergence of cancer itself). In this way, carcinogenesis has been interpreted as a loss of information in repair mechanisms, and cancer metastasis has been associated with an increase in entropy in protein–protein interaction networks (Figure 1A) [12,27–29]. Entropy can also serve as a validation measure in the computational tool; for this purpose, the ImmunoMap was created to identify T-cell receptor signatures as clinical biomarkers in pre- and post-therapy samples [28].

A natural question that arises from the quantification of entropy is the interpretation of the maximum entropy (ME) state in a system. In the context of immune system function, ME was applied to signaling network data from the epidermal growth factor receptor (EGFR)/Akt signaling pathway and resulted in the computational tool ME-based fRamework for Inference of heterogeneity in Dynamics of sIgnAling Networks (MERIDIAN) [29]. The MERIDIAN framework was used to investigate heterogeneity in cell signaling networks by analyzing the joint probability distribution of parameters in a mechanistic model of cell signaling networks. The MERIDIAN analysis was found to be consistent with experimentally observed cell-to-cell variability of phosphorylated Akt and cell surface EGFR expression and was able to predict an ensemble of single-cell trajectories for different time intervals and experimental conditions (Figure 1B). Taking the concept of ME even further, it was suggested that, because no living system is in thermodynamic equilibrium, all living systems require their information content to be maintained at an extremum to maintain stable entropy [30]. This ‘evolutionary’ argument suggests a transition from information minima for lower organisms to maxima for higher organisms; in contrast, carcinogenesis can be viewed as a reverse transition from an information maximum to minimum.

### Mutual Information for Heterogeneous Datasets and Variable Types

In the case of complex datasets, evaluation of dependency between two or more mixed-type variables requires further generalization of entropy measures. Mutual information, a measure where a distribution of *x* can be drawn from a conditional distribution, instead of that of an individual, is used frequently in multivariate immune system studies [20]. The concept of mutual information does not diverge far from ‘simple’ entropy changes; the measure of information about *x* gained by observing *y* can be reflected through a reduction in entropy.

Notably, mutual information is an intrinsically flexible measure that can be extended to heterogeneous variables and mixed variable types (e.g., spatially defined variables). Pointwise mutual information (PMI) was used to identify biomarker patterns and cellular phenotypes derived from breast cancer immunofluorescence pathology samples [31]. PMI helped quantify intratumor heterogeneity scores by comparing spatial maps of PMI from patients across cancer types and subtypes. While using IT-based approaches, the potential of PMI to quantify tumor heterogeneity from previously unstudied elements of the tumor microenvironment (e.g., non-cellular constituents) was demonstrated. In another application of mutual information to spatial data, T-cell activation in pathogenic infections was characterized by applying normalized mutual information measures with the Pearson correlation coefficient to determine the extent to which naive T cells associated with dendritic cells, fibroblastic reticular cells, and blood vessels within lymph nodes [32]. In this case, normalizing mutual information allowed the authors to directly compare mutual information across experiments and to gain insight into factors that drive T-cell localization and interactions between T cells and dendritic cells.

Mutual information has also been used to interrogate cytokine signaling networks in immune cells. A computationally efficient analysis of biochemical signaling networks such as cytokines and intracellular networks using Statistical Learning-based Estimation of Mutual Information (SLEMI) (Figure 1C) was proposed in [33,34]. The use of mutual information in SLEMI allowed the investigators to inter-relate a large number of inputs and high-dimensional outputs, which previously were limited to single-input–single-output analyses. Such a computational advance enables an information-theoretic study of signal transmission and processing in cells with complex high-dimensional datasets and extends to calculations of information capacity [35]. By integrating a mutual information approach with kernel density estimators, mutual information was used to rank and identify significant components of phosphoprotein-cytokine signaling networks for several cytokines [36].

Mutual information has also been used in a clinical context. A multivariate mutual information algorithm was developed in [37] to match patients with ovarian cancer to potential therapies. The novel tool, CorEx, was used to stratify patients for survival analysis through the use of RNA-sequencing profiles (Figure 1D). By maximizing mutual information to infer complex hierarchical gene expression relationships directly from transcription levels, these findings support the use of IT-based metrics for the selection of personalized and effective cancer treatments. Similarly, another integrated mutual information-based network inference approach was introduced: an Algorithm for the Reconstruction of Accurate Cellular Networks (ARACNE) [38]. By using ARACNE, the authors successfully used weighted integration of IT measures in nearly 500 breast cancer samples to detect and identify modules in cancer subtype networks (luminal A, luminal B, basal, and HER2 enriched) [39].

In general, due to the recent progress in the mutual information methodological research [18,36,40–42], application of mutual information to the biomedical datasets is relatively straightforward and flexible. However, one should be mindful of fine-tuning mutual information estimation parameters, such as the number of neighbors in the *k*-nearest neighbors mutual information estimator [43], which ideally should be carried out *de novo* for each new dataset. Similarly, there is an issue of the proper weighting of ‘subjective’ (i.e., expert-driven) importance of different data types. While the above complication is more of a feature construction (than pure IT) issue, it, again, should be investigated *de novo* for each new dataset. Having acknowledged that, mutual information can be seamlessly integrated into comprehensive analytical frameworks generalizable to many biomedical data analysis scenarios. In our own experience, mixed multivariate mutual information is a reliable and consistent measure in variable selection and Bayesian network modeling [44]. Examples of available IT software packages and tools are summarized in Box 2.

### Measures of Divergence between Distributions

Probability distance measures and metrics [e.g., KL divergence, JSD, earth mover’s (EM) distance, Kolmogorov-Smirnov (KS) distance] are often derived from, or analogous to, relative entropy and cross-entropy measures. They have been used to measure distributional similarity to improve probability estimation for unseen co-occurrences and to quantify distributional dissimilarity across many domains in biology [26,45–47]. Immuno-oncology *per se*, however, has not encountered many applications to date; therefore, here we outline existing cancer- or immunology-related applications, with an eye toward future perspectives for immuno-oncology.

One prominent example of distributional measures in immuno-oncology is in the analysis of gene mutations or methylation states. Rather than compare individual loci in the genome, one may ask a more general question: Are the distributions of mutations or methylation profiles across a wide range of the genome different, and do these differences carry an information-theoretical interpretation? In an analysis of DNA methylation, KL divergence and JSD were applied simultaneously to discriminate between differentially methylated genes in healthy and tumor samples [40]. Integration of KL divergence or JSD into machine learning analytical frameworks has been employed in prediction of plasma samples using microsatellite status as a biomarker and in single-cell gene expression analyses to trace the transcriptional roadmap of individual CD8^+^ T lymphocytes [48,49]. Whereas EM distance could predict biomarker expression levels in cell populations from flow cytometry data, the KS distance showed applicability to one-dimensional samples in two-sample testing cases; as such, KS became one of the most popular measures for two-distribution comparisons [47]. A few recent translational applications of IT-related distance metrics include prediction of gut microbiome–mediated response to immunotherapy in patients with melanoma [50], quantification of immune cell subtypes from histological samples [48], and estimation of T-cell receptor repertoire divergence in patients with glioblastoma [49].

## Information Flow and Signal Processing: From Communication Channel to Cellular Interactions

Because the immune system relies on efficient and accurate transmission of information, there is inherently a temporal dynamic to immune function and response. We note that entropy, mutual information, and distributional metrics are not commonly defined in a time-dependent manner; below we discuss the temporal aspects [51] of immune signaling and flow of information through the immune system as an extension of the ‘static’ IT framework.

### Communication Channel and Its Capacity

Signaling pathways within and between cell populations of a healthy or tumor-affected immune system create a communication network that involves encoding, communicating, and decoding information between sender(s) and receiver(s) through a communication channel (Box 1). Such a system is composed of upstream and downstream molecules, with their concentrations dictating the patterns of interaction. Here, we review the concept of a communication channel, representative of the complexity of signal flow between a sender and receiver. We follow with a discussion on the intrinsic or extrinsic noise affecting its capacity.

The representation of input–output dependability within the communication channel of signaling cascade from tumor necrosis factor (TNF) receptor to nuclear factor-κB (NF-κB) and collective dynamics of cell responses to increase information transfer in a noisy environment [52]. This ability to mitigate noise in order to maintain information gain and channel capacity was applied to extracellular signal-regulated kinase (ERK) dynamics [43]. Moreover, the intermediate states existing in the ERK communication channel can possess a dual sender/receiver nature [53]. For example, growth factor–mediated phosphorylation of ERK generates distinct patterns of concentrations that control gene expression levels and cell fate. Whether the kinase is a sender for downstream gene expression or a receiver for upstream growth factors depends on the actual question. In an example discussed in [16], cytokines signal to the cell nucleus through a network of transcription factors, which possess a dual sender/receiver nature. Inevitably, the overall transmission process is not perfect; transcription factors can only carry as much of the information as can be received by the downstream protein encoders. Thus, the accuracy of a response depends on the amount of information lost via signal transfer from cytokine to transcription factors and then to the nucleus.

### Communication in Noisy Environments

Information gained about an input (signal) by measuring its noisy representation (response) can be quantified through reduction of uncertainty using mutual information (Equation 3). Mutual information allowed investigators to explicitly quantify transmitted information and thus estimate the channel capacity of the signaling pathways in crowded environments; consequently, direct approaches to quantifying dynamic systems in immuno-oncology have begun to emerge [33–35,43].

Estimating information flow becomes more difficult with an increasing number of signaling molecules exposed to diverse sources of noise [54]. Various concentrations of signaling molecules generate unique patterns, which can control cell fate in different ways. This process can be affected by internal (e.g., inherent stochasticity of biochemical process) and external factors of a random nature (e.g., variations in the expression levels) sensed by each cell [51,43,55]. In the simplest case, when a single signal is sent from one molecule to another with no intermediate players, information transfer can only be affected by noise [16,43,56]. Assessing the level of noise is critical for accurately measuring information transmission between the ends of a signaling channel. IT methods provide tools to estimate the impact of noise (e.g., initial concentrations of signaling cells, variations in the environment); yet, the reliability of signal transduction measures is reduced for populations of cells. In reality, signaling molecules emit and detect multiple signals simultaneously, which in turn may cause signal overlap. If the distributions of these signals do not overlap, the receiver will recognize them as independent signals transmitted between the upstream and downstream molecules and allow a ‘perfect’ estimation of mutual information. If, however, the signals overlap, accurate estimation of mutual information decreases because the signals cannot be separated. More details on the noise decomposition within the biochemical signaling networks using the ‘noise mapping’ technique can be found in [57].

To this end, the intrinsic molecular variability was defined as the thermodynamic-in-nature noise of molecular interactions, which requires stochastic mathematical representations and may limit the predictability of biological phenotypes or signal transduction reliability [55,58]. On the contrary, extrinsic noise reflecting different starting conditions can be defined [43,55] and modeled using deterministic (i.e., ordinary differential equations) [54] or stochastic [59] mathematical models. Using the former, the effects of noise on mutual information in signaling pathways (input–output, linear, and feedforward loop motifs) were evaluated under the effects of extrinsic, intrinsic, or both types of noise [54]. The reduction of uncertainty, through mutual information, associated with potential outcomes was the most impacted by extrinsic noise, when mutual information between input and output was maximal. The authors suggested that information transmission is most affected by intrinsic noise across all three motifs. The application of dynamic stochastic differential modeling over time allowed the analyses to be carried out for entire trajectories of signaling systems. In the latter study [59], the effects of intrinsic molecular noise associated with the number of activated receptors under various stimulative conditions allowed researchers to identify the sensitivity and limits of information transfer for the NF-κB pathway, whereas the extrinsic noise due to the variability in cellular states was associated with discernibility of a dose and changes in mutual information.

### Information Flow between Correlated and Uncorrelated Variables over Time

Given that a single cell may be exposed to many sources of information that may fluctuate over time, time integration within collective cell responses was considered as an explanation for the increase in information transfer [52]. This integration was proposed as a solution to the observed ‘bottleneck’ noise (restrictions in cell response due to noise) that can significantly restrict the amount of information within signaling pathways. The authors presented ‘bush and tree’ network models as a framework for analyzing branched motifs. Using this approach, they discovered that receptor-level bottlenecks restricted communication in the TNF and platelet-derived growth factor networks, an observation likely to be prevalent in other signaling systems.

Thus far, we have discussed applications of IT measures to data collected over time. Another approach is to use dynamic mathematical models to predict the temporal evolution of the system, which can facilitate IT measurements of information transfer within dynamic systems. To this end, the formalism of information transfer was proposed within stochastic dynamic systems, which might be applicable to information flow in the immune system [60–64]. The authors proposed the need to measure the entropy rate transferred from one component to another, stating that even for two highly correlated time series, there could be no information transfer between the variables. The authors demonstrated the existence of hidden information in high-dimensional systems; such indirect components could help explain information flow in the immune system, in which many variables that may be correlated may in fact not be transferring information.

### Potential Applications of Information Theory Concepts to Communication within the Immune System

Within the complex and interdependent networks and pathways of the immune system, IT concepts, especially mutual information, offer a robust and multifunctional measure of dependencies and quantification of information between variables. Combining mutual information with communication channels and channel capacities allows comparisons across different settings and conditions, for example. Current applications of IT-derived concepts are summarized in Table 1. Here we present potential applications of IT concepts to immuno-oncology based on successes from other biomedical fields.

**Figure 2.**
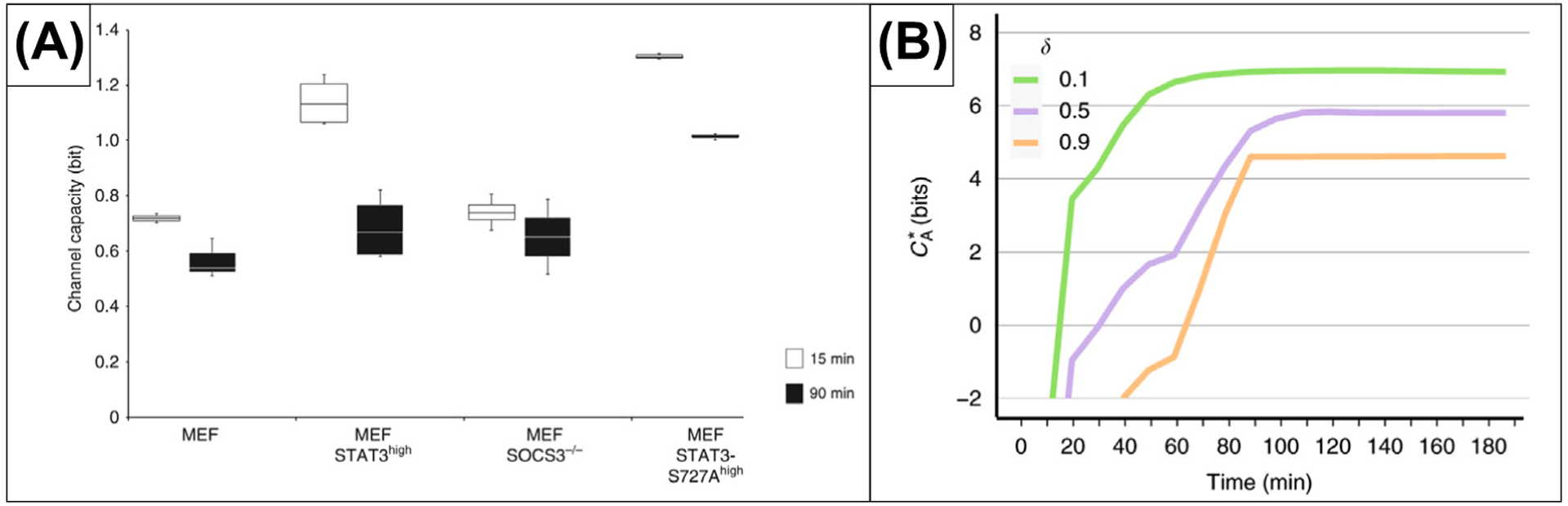
Information Theory Approaches to Calculations of Channel/Information Capacity in Signaling Pathways. (A) Channel capacity of JAK/STAT signaling at 15and 90 min after induction by cytokine interleukin (IL)-6. Four immortalized murine embryonal fibroblast cell populations were analyzed from left to right: (i) wild type, (ii) with high STAT3 expression, (iii) feedback-inhibitor suppressor of cytokine signaling 3-deficient, and (iv) carrying serine-to-alanine mutation. Data are from *n*=3, 4, 4, and 3 independent experiments, respectively. Reprinted, with permission, from [34] under the Creative Commons license http://creativecommons.org/licenses/by/4.0/. (B) Transfer of information by signaling dynamics of interferon (IFN)-α and IFN-λ1. Information capacity $\left(C_{\text{A}}^{*}\right)$ for different values of the differential kinetics coefficient, δ. Reprinted, with permission, from [35] under the Creative Commons license (as earlier). Abbreviations: MEF, mouse embryonic fibroblasts; SOCS: Suppressor Of Cytokine Signaling

Besides comparisons between tumor versus normal tissue, various cell lines or pathways among dynamic systems, and temporal applications, promising applications arise from studies of phenotypes originating from mutations or phosphorylation spatial heterogeneity [34] (Figure 2A). In the context of the immune system, channel capacity can be seen as a generalization of statistical comparisons in multidose settings; that is, the calculations of the number of bits of the system or the number of distinguishable states can give the comprehensive measure of sensitivity of given conditions expressed by a dose–response curve [65]. Analogous to what was previously shown in the TNF–NF-κB signaling cascade, where quantifying the system’s ability to discriminate nearby concentrations was achieved by estimating the upper bound of mutual information [59], turning the subsequent steps of signaling pathways into a binary decision could assist in deconvolution of complex cytokine networks. Optimization of channel capacity can be an appreciated tactic to predict the environmental distribution of the intra- or extracellular signals and transcriptional regulation [66,67]. Collective behavior of cell populations [35] (Figure 2B) and aggregation of information can be confronted by interpretations of the cell subpopulations processing information independently [68]. Aspects of information transfer in signal transduction on the levels of single cell vs. population [65,69] could be a promising approach to control cell death in immunotherapies, considering apoptosis as the key physiological variable defining the fraction of cells responding to a given dose.

### Concluding Remarks

We have highlighted many aspects of information theory and information flow within the immune system, from molecules to cells and populations of cells. However, the rules that govern immune system intercellular communication remain poorly understood. A major hurdle comes from the complexity of intrinsic and extrinsic interactions that single molecules experience; thus, cellular processes involving signal detection, communication, and transmission are not a fully understood phenomenon at this time [70]. Given recent technological advances in biological data collection at multiple scales in space and time, we believe that, in the foreseeable future, our ability to model information flow and signal processing in the immune system is likely to be advanced by a deeper integration of existing IT and experimental tools (see Outstanding Questions), augmented by crosstalk between theoretical models [71]. In our opinion, the wide variety of applications of IT to the immune system foretell a much wider acceptance of IT concepts and methods in immuno-oncology in the immediate future.

## Figures and Tables

**Table 1.** Definitions, Meanings, and Applications of Information Theory Concepts to the Study of the Immune System and Immuno-Oncology^b^

Concept	Mathematical definition	Meaning	Application	Refs
Entropy, maximum entropy	*H*(*X*) = − ∑_*x*∈*X*_*P*(*x*) log *P*(*x*)	System organization/disorganization	T-cell receptor diversity Information capacity EGFR/Akt signaling Carcinogenesis	[28] [35] [29] [28–30]
Mutual information	*I*(*X*;*Y*) = *H*(*X*) − *H*(*X*|*Y*) $H\left(X|Y\right)=-\sum_{x\in X,y\in Y}P\left(x,y\right)\text{ log}\frac{P\left(x,y\right)}{P\left(y\right)}.$	Information shared between variables	Biomarker cellular patterns T-cell activation and spatial organization in lymph nodes Cytokines and protein interaction networks Machine learning	[31] [32] [33,34], [36] [44]
Cross and relative entropy	$CE\left(P\|Q\right)=\sum_{x\in X}P\left(x\right)\text{log}\frac{P\left(x\right)}{Q\left(x\right)}\;-\sum_{x\in X}P\left(x\right)\text{ log }P\left(x\right)$	Information shared between distributions of variables	Biomarker identification Comparison of transcriptional states (e.g., CD8^+^ vs. CD4^+^) Machine learning	[40] [42] [60–63] [41]
Channel capacity^a^	$C=\underset{P_{X}(x)}{\text{sup}}I\left(X;Y\right)$	Maximum rates at which information can be reliably transmitted over a communication channel	JAK/STAT signaling Cytokine signaling, gene expression Intrinsic and extrinsic noise in signaling	[34] [16,53] [54,58]
Information transfer and flow^b^	$\frac{d\text{x}}{dt}=\text{F}(x,t);\frac{dH}{dt}=E(\nabla \cdot \text{F})$	Transfer of information between correlated or uncorrelated variables over time	Spatial and temporal dynamics Information flow in dynamic systems	[60–63] [73]

a Sup is the supremum, or least upper bound of mutual information *I(X;Y)* over the marginal distribution *P*_*X*_*(x)*.

b ***F*** is a vector field of a dynamical system (d***x***/d*t*), d*H*/d*t* is the evolution of entropy (*H*), equal to the expectation (*E*) of the divergence (∇) of the vector field ***F***.

## Highlights

Information theory (IT) may be used to define and guide new concepts in the study of immune signaling in health and in cancer.

IT concepts are well suited for understanding biochemical signaling and cellular dialogues in the immune system in terms of information flow, signal processing, and communication channels.

Cancer research can benefit from specific applications of IT to elucidate immune system defects in cancer, and, subsequently, cancer therapies can benefit from IT-informed modulation of the immune response.

Given recent advances in sequencing technologies and the prevalence of immune-modulating therapies, we anticipate that IT concepts and methodologies will become a prominent trend in cancer research in the near future.

## Box 1. Historical View of Information Theory

Information Theory (IT) describes and quantifies information storage and communication in a mathematically rigorous fashion. IT was originally proposed to address limits and loss of signal processing in data transfer by Shannon and Weaver [1,2]. Shannon theory, or the Shannon–Weaver model, was aimed at integrating and formalizing all components of a generalized communication system. Entropy is a central measure in IT and quantifies uncertainty associated with the outcome of a random process. Other important measures include mutual information, communication channel, and channel capacity, with mutual information reflecting the mutual dependence between variables by amount of information gained by observation of one variable through another variable. A communication channel is an IT-derived model of information flow over time between variables in a dynamic system (Figure I). One property of a communication channel is its capacity, which is directly linked to unavoidable loss of information due to environmental noise, such as within signaling systems. In the context of immune system communication, noise (intrinsic, extrinsic, or a mixture of both) can negatively contribute to the capacity of a signaling channel; noise disrupts precise signaling and naturally differs between cell types and associated communication patterns. These concepts from information theory apply to the immune system through signal transduction and transmission of information in signaling pathways.

## Box 2. Software and Packages for Applications of Information Theory Concepts in Practice

Recently, several software packages have become available with valuable up-to-date methods to calculate IT-related quantities, such as entropy, mutual information, and channel capacity. Here we focus on the tools relevant to signaling pathways or communication between signaling molecules within the immune system.

The Maximum Entropy-based fRamework for Inference of heterogeneity in Dynamics of sIgnAling Networks (MERIDIAN) [29] is an open source MATLAB package (https://github.com/dixitpd/MERIDIAN). The Statistical Learning-based Estimation of Mutual Information (SLEMI) package (Figure 1C) provides application of mutual information and channel capacity calculations for molecules active within the JAK/STAT signaling pathway that is not limited to single-input–single-output analyses but allows the analyses of complex high-dimensional datasets. More on SLEMI applications of the open source R package in CRAN (https://github.com/sysbiosig/SLEMI) can be found in [33,34]. The R and MATLAB codes for channel capacity calculations within the interferon signaling pathway [35] are available at: https://github.com/sysbiosig/Jetka_et_%20al_Nature_communications_2018. The open source package used for the reconstruction of the cellular networks using a mutual information-based interference approach applied to breast cancer from [38] is available at https://github.com/califano-lab/ARACNe-AP. The *k*-nearest-neighbor approach also supports mutual information calculations and estimation of channel capacity within mitogen-activated protein kinase pathway (https://github.com/pawel-czyz/channel-capacity-estimator). Multilevel mutual information estimates under conditions confronting small sample size, long time-series trajectories, and time-varying signals with extrinsic noise can be found in [72] for mammalian intracellular signaling.

Due to the focus of this review on the immune system and immuno-oncology, many applications and mathematical approaches up to date for information estimation and other packages and from other fields are not discussed here but can be found at: http://strimmerlab.org/software/entropy/ (entropy R package); https://github.com/maximumGain/information-theory-tool (MATLAB source code for basic computations in IT); https://github.com/cran/infotheo (infotheo R package); https://github.com/robince (IT quantities from neuroscience, including discrete, univariate continuous, multivariate continuous correlation analyses); https://github.com/NeoNeuron/tdmi (time-delayed mutual information for neurophysiology data C/C++ package).

## Outstanding Questions

How can information theory (IT) be used to discover new and understand known defective signaling patterns in the immune system in cancer?

How will IT approaches help fill the gap in our understanding of the immune system to improve the development of immune-modulating therapies in cancer?
